# Comparison of therapeutic dosimetric data from passively scattered proton and photon craniospinal irradiations for medulloblastoma

**DOI:** 10.1186/1748-717X-7-116

**Published:** 2012-07-24

**Authors:** Rebecca M Howell, Annelise Giebeler, Wendi Koontz-Raisig, Anita Mahajan, Carol J Etzel, Anthony M D’Amelio, Kenneth L Homann

**Affiliations:** 1 Department of Radiation Physics, The University of Texas MD Anderson Cancer Center, Houston, TX, USA; 2 Graduate School of Biomedical Sciences, The University of Texas at Houston, Houston, TX, USA; 3 Department of Radiation Oncology, The Emory Clinic, Atlanta, GA, USA; 4 Department of Radiation Oncology, The University of Texas MD Anderson Cancer Center, Houston, TX, USA; 5 Department of Epidemiology, The University of Texas MD Anderson Cancer Center, Houston, TX, USA; 6 Department of Radiation Physics, Unit 094, The University of Texas MD Anderson Cancer Center, 1515 Holcombe Blvd, Houston, TX, 77030, USA; 7 Present Address: Louisiana State University, Department of Physics and Astronomy, Baton Rouge, LA, USA

**Keywords:** Proton, Photon, Craniospinal irradiation, CSI, Medulloblastoma

## Abstract

**Background:**

For many decades, the standard of care radiotherapy regimen for medulloblastoma has been photon (megavoltage x-rays) craniospinal irradiation (CSI). The late effects associated with CSI are well-documented in the literature and are in-part attributed to unwanted dose to healthy tissue. Recently, there is growing interest in using proton therapy for CSI in pediatric and adolescent patients to reduce this undesirable dose. Previous comparisons of dose to target and non-target organs from conventional photon CSI and passively scattered proton CSI have been limited to small populations (n ≤ 3) and have not considered the use of age-dependent target volumes in proton CSI.

**Methods:**

Standard of care treatment plans were developed for both photon and proton CSI for 18 patients. This cohort included both male and female medulloblastoma patients whose ages, heights, and weights spanned a clinically relevant and representative spectrum (age 2–16, BMI 16.4–37.9 kg/m2). Differences in plans were evaluated using Wilcoxon signed rank tests for various dosimetric parameters for the target volumes and normal tissue.

**Results:**

Proton CSI improved normal tissue sparing while also providing more homogeneous target coverage than photon CSI for patients across a wide age and BMI spectrum. Of the 24 parameters (V_5_, V_10_, V_15_, and V_20_ in the esophagus, heart, liver, thyroid, kidneys, and lungs) Wilcoxon signed rank test results indicated 20 were significantly higher for photon CSI compared to proton CSI (p ≤ 0.05) . Specifically, V_15_ and V_20_ in all six organs and V_5_, V_10_ in the esophagus, heart, liver, and thyroid were significantly higher with photon CSI.

**Conclusions:**

Our patient cohort is the largest, to date, in which CSI with proton and photon therapies have been compared. This work adds to the body of literature that proton CSI reduces dose to normal tissue compared to photon CSI for pediatric patients who are at substantial risk for developing radiogenic late effects. Although the present study focused on medulloblastoma, our findings are generally applicable to other tumors that are treated with CSI.

## Background

Medulloblastoma is the most common malignant childhood brain tumor. In recent decades, the 5-year survival rate for this cancer has improved from 60% to between 80% and 85% for average-risk patients and from 35% to between 60% and 70% for high-risk patients [1-3]. The primary tumor normally originates in midline cerebellar structures with infiltration of surrounding posterior fossa and may disseminate throughout the neuroaxis via cerebrospinal fluid (CSF) pathways [4-6]. Treatment for medulloblastoma thus often includes chemotherapy and craniospinal irradiation (CSI) [7-9], including a boost to the posterior fossa or the surgical bed with a margin.

Because of the high survival rate, the fact that patients require radiotherapy, and the fact that children and adolescents are more likely to develop radiation-related late effects than adults, late (>5 years after treatment) effects from radiation are a major concern for medulloblastoma patients [10-13]. The late effects associated with CSI are well-documented and may include (but are not limited to) impaired growth [14], endocrine abnormalities [15-17], hearing loss [17,18], diminished fertility [17], neuropsychological dysfunction [17,19], cardiac diseases [17,20-24], and second cancers [17,22-28]. For many decades, the standard of care radiotherapy regimen for CSI has been photon (megavoltave x-rays) therapy that included opposed lateral cranial fields and either single or multiple posterior spinal fields [29]. Late effects are, in part, a consequence of dose from the CSI treatment fields to various non-target organs. Compared to photons, protons have substantially lower entrance dose and almost no exit dose and thus can significantly reduce the dose to all organs situated outside the craniospinal axis which are irradiated unnecessarily. Consequently, there is growing interest in using proton therapy for CSI in pediatric and adolescent patients.

Passively scattered proton CSI has been shown to improve dose uniformity along the spinal canal and decrease dose to non-target organs compared with photon CSI [30-33]. However, each of these studies was limited to a very small number of patients—there were a total of 7 patients, 6 of whom were under the age of 5, in all the studies combined—limiting the results’ applicability and understanding of dosimetric differences across a wide spectrum of patient ages and body sizes. Finally, none of these reports addressed the differences in target volumes used in planning proton and photon CSI, e.g., the age-specific target volumes used in proton CSI. Thus, we sought to carry out a detailed comparison of the current treatment standards for photon and proton CSI for a population of both male and female medulloblastoma patients whose ages, heights, and weights spanned a clinically relevant and representative spectrum (age 2–16, BMI 16.4–37.9 kg/m^2^) with a focus on the differences and variations in target volume definition and dose delivered between photon and proton therapy.

## Methods

### Study patients

This study was carried out under a protocol for retrospective treatment planning studies approved by our institution (University of Texas at M.D. Anderson Cancer Center, UTMDACC). We compared therapeutic dose distributions for photon and proton CSI for a group of 18 consecutive patients (8 girls and 10 boys). The inclusion criteria were that the patients be between 2 and 18 years old at the time of treatment and were treated with proton CSI at our institution between 2007 and 2009. The patients in this study had a mean age of 9.5 years (range, 2–16 years). Patient age, sex, height, weight and BMI are listed in Table 1.

**Table 1:** Patient characteristics

**Index**	**Age**	**Sex**	**Height (cm)**	**Weight (kg)**	**BMI (kg/m**^**2**^**)**
1	2	female	85.0	11.9	16.5
2	4	female	111.7	20.5	16.4
3	6	female	115.2	26.9	20.3
4	8	female	142.0	37.5	18.6
5	10	female	130.6	24.2	14.2
6	2	male	109.2	18.9	15.8
7	4	male	128.0	31.3	19.1
8	6	male	144.8	24.9	11.9
9	8	male	123.4	20.3	13.3
10	10	male	133.0	28.2	15.9
11	12	female	146.0	28.9	13.6
12	13	female	-------------------------------------- data not available ---------------------------------		
13	16	female	162.0	62.0	23.6
14	12	male	166.3	66.5	24.0
15	13	male	173.0	57.5	19.2
16	14	male	162.5	58.6	22.2
17	15	male	172.1	73.3	24.7
18	16	male	191.0	138.2	37.9

Patients underwent computed tomography (CT) simulation while in the supine position with their heads immobilized using an Aquaplast face mask (WFR/Aquaplast Corp. and Qfix Systems, LLC, Avondale, PA) and a plastic head holder to reduce kyphotic neck curvature. The CT images were acquired on a multi-slice CT scanner (General Electric (GE) LightSpeed RT16, GE Healthcare, Waukesha, WI) and had a 2.5-mm slice thickness.

Both photon and proton treatment planning were carried out according to the standards of care at our institution (UTMDACC). We streamlined plan comparisons by using the same commercial treatment planning system (TPS) for both modalities (Eclipse version 8.9, Varian Medical Systems, Palo Alto, CA). All treatment plans were calculated using a 2.5-mm calculation grid with heterogeneity corrections. Dose distributions in the photon and proton plans were respectively calculated using anisotropic analytical and pencil beam algorithms. The proton calculation algorithm was previously validated using the methodology described by Newhauser et al. [34] and the photon algorithm was commissioned following methodologies described in the literature [35,36]. The beam arrangements for the photon and proton treatment plans were similar. Both included two opposed lateral oblique cranial fields, which were angled so that they avoided ocular structures, and postero-anterior spinal field(s). The proton plans used one to three spinal fields while the photon plans used either one or two spinal fields to cover the entire length of the spinal canal through the inferior extent of the thecal sac, typically at the level of the S2/S3 vertebral junction. The spinal fields were matched at the posterior edge of the vertebral canal (not on the vertebral body).

**Figure 1:**
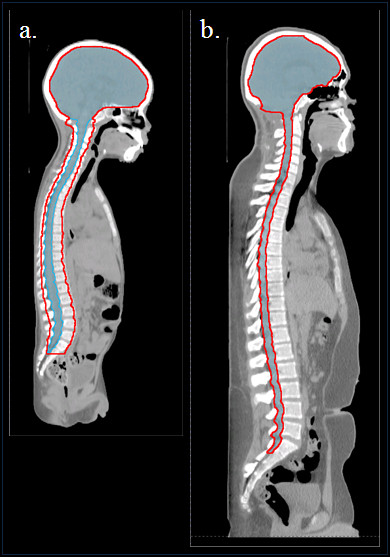
** Age-specific target volumes for proton treatment planning (red contour) and CTVs (blue color wash) in proton and photon treatment planning for representative patients.** (**a**) Volumes for a 4-year-old patient and (**b**) volumes for a 15-year-old patient.

The total prescribed dose was 23.4 Gy relative biological effectiveness (RBE) (i.e., 21.3 Gy × 1.1 to reflect the biological effectiveness of protons relative to photons) and 23.4 Gy for the proton and photon CSI treatment plans, respectively. Hereafter, dose units will be simply be referred to as Gy and Gy or Gy-RBE for photons and protons, respectively. The use of the generic RBE factor of 1.1 is in accordance with the recommendations on dose prescription and reporting in International Commission on Radiation units and Measurements (ICRU) Report 78 [37]and consistent with the clinical practice at our institution. However, it noted that the recommended RBE value has never been measured in humans who received proton therapy [37]. The prescription dose of 23.4 Gy was selected for this study because it the most commonly used dose for moderate risk patients and is the dose used at our institution for such patients. However, for high risk patients the CSI dose can be as high as 36 to 39.6 Gy and but may also be as low as 18 Gy, which is currently being evaluated by some institutions. The fractionation schedule was 1.8 Gy per fraction for 13 fractions with 2 junction shifts (initial and 2 shifted positions), which is a common dose and fractionation pattern for patients with average-risk medulloblastoma. The clinical target volume (CTV) for both the photon and proton treatment plans included the entire CSF space (the brain and spinal canal through the cauda equina to the level of the S2/S3 vertebral junction (Figure 1). Additionally for patients under the age of 15 years there was an additional target volume which was also treated to the full prescription dose (discussed below in the section on proton therapy planning). All treatment plans were reviewed by a board certified medical physicist (R. Howell) and reviewed and approved by a board-certified radiation oncologist who specializes in pediatric radiotherapy (A. Mahajan).

### Proton therapy treatment planning

In this study (and in accordance with clinical practice at our institution), we used age specific target volumes for proton CSI treatment planning. For all proton CSI patients, the CTV included the entire CSF space (the brain and spinal canal through the cauda equina to the level of the S2/S3 vertebral junction [Figure 1) and was equivalent to the photon CTV. Additionally, for patients under the age of 15 years there was an additional normal tissue target volume (NTTV), which included the entire vertebral bodies. The rationale for this was to avoid sharp dose gradients in the vertebral bodies in patients whose skeletons were still maturing. More specifically, proton treatments that are designed to irradiate only the spinal canal have high dose gradients distal to the spinal canal and lead to non-uniform irradiation of the vertebral bodies. Uniformly irradiating a larger target volume that fully encompasses the vertebral bodies is thought to reduce the risk of asymmetric growth of the vertebral body in patients whose skeletons are still maturing [33,38] i.e., those under the age of 15 years.

Adequate uncertainty margins are especially important in proton therapy because proton fields are especially sensitive to patient positioning due to several factors including: 1) proton fields have a sharp distal fall-off, but the location of that fall-off is dependent on the beam range which is determined by the composition of tissues in the beam path; thus, lateral or superior/inferior shifts in patient position, relative to the field’s isocenter, can change the location of the distal field edge relative to specific organs of interest and 2) proton fields are shaped by field specific apertures and tissue compensators, so lateral or superior/inferior shifts in patient position, relative to the field’s isocenter, can shift patient anatomy from its optimal alignment to these devices. To ensure the proton treatment fields had appropriate uncertainty margins we used the methodology of the ICRU Report 78 [37]. As a result, field parameters were determined using the CTV, rather than the PTV, and the burden of applying the parameters was placed on the computer algorithm. That is, values for compensator smear, lateral, proximal, and distal margins were manually calculated for each beam using a methodology similar to that used in our previous studies [39,40] and following the methods originally outlined by Urie et al. [41] and Moyers and Miller [42] and Moyers et al. [43]. Once calculated those values were entered into the TPS as planning parameters. Then, the TPS selected the corresponding machine parameters (beam energy, range modulation, and range shifter settings), designed the compensator, and sized the apertures. For patients older than 15, these uncertainty margins were designed to ensure coverage of the CTV. Similarly, for patients younger than 15 the uncertainty margins were designs such that the CTV as well as the entire vertebral bodies (NTTV) received the full prescription dose.

Beam energies for the proton plans were patient and field specific and included energies of 140 MeV, 160 MeV, 180 MeV, 200 MeV, and 225 MeV. The mean cranial and spinal field energies were 198 MeV (SD = 12 MeV) and 163 MeV (SD = 17 MeV) for the cranial and spinal fields, respectively. The mean range was 17 cm (SD = 1 cm) and 11 cm (SD = 2 cm) for the cranial and spinal fields, respectively. The mean Spread out Bragg peak was 16 cm (SD = 1 cm) and 5 cm (SD = 1 cm) for the cranial and spinal fields, respectively. A more comprehensive and detailed description of the proton CSI treatment planning technique used in this study is reported in the literature by Giebeler et al. (in review).

### Photon treatment planning

The photon CSI plans were calculated using a beam energy of 6 MV. After the cranial and spinal field geometries [29,44] were defined, multiple lower-weighted reduction fields within the primary cranial and spinal fields were added to minimize dosimetric heterogeneities (reduce hot spots in thinner regions of the anatomy and cold spots in thicker regions of the anatomy). The reduction fields contained blocked segments strategically placed to reduce the highest dose areas to force greater homogeneity and conformity in the target volume. This planning technique is commonly referred to as intensity-modulated field-in-field planning and was described in detail by Yom et al. [45]. Photon treatment plans were normalized so that the 100% isodose line covered the CTV and allowed for setup-up uncertainty.

### Comparison of photon and proton treatment plans

We compared three dosimetric parameters for the CTV: the maximum dose (D_max_), the conformity index (CI), and the heterogeneity index (HI). The CI is defined as

(1) $$CI=\frac{V_{\mathrm{Rx}}}{V_{\mathrm{EV}}}$$

where V_Rx_ is the volume receiving the prescribed dose and the V_EV_ the total CTV and HI is defines as

(2) $$HI=\frac{D_{5\%}}{D_{95\%}}$$

where D_5%_ is the dose delivered to the hottest 5% of the CTV and D_95%_ is the minimum dose received by 95% of the CTV.

The HI was used to quantify dosimetric homogeneity within the CTV. A lower HI indicated a more uniform dose distribution. The CI was used to quantify how well the prescribed dose conformed to the CTV. A lower CI indicated a more conformal dose distribution.

In addition to the CTV, we contoured the following normal tissues so we could compare photon and proton doses in organs that were within or near the treatment fields: spinal cord, optic chiasm, cochlea, brainstem, esophagus, heart, kidneys, liver, lungs, and thyroid. A dose volume histogram (DVH) was calculated for each of these structures. Then, we quantitatively compared the photon and proton DVH data for each structure by comparing the mean percent volume (V) receiving various specified dose levels in units of gray (Gy). V_23.4_ and V_25_ were compared for the CTV and organs that were entirely within the treatment fields. V_5_, V_10_, V_15_, V_20_, and V_23.4_ were compared for partially in-field and out-of-field organs.

### Statistical methods

Statistical analyses were performed to compare the various dosimetric parameters for the CTV and the normal organs. We used the Wilcoxon signed rank test with a null hypothesis that the differences between the various dosimetric parameters for photon and proton therapy come from a continuous, symmetric distribution with zero median. For the CTV and organs entirely within the CTV (optic chiasm, cochleas, brainstem, spinal cord), we used a two-tailed Wilcoxon signed rank test to compare these values. The alternative hypothesis for this two-tailed test was that the differences between the various dosimetric parameters for photon and proton therapy come from a continuous, symmetric distribution with a positive or negative median. For partially in-field and out-of-field organs (esophagus, heart, kidneys, liver, lungs, and thyroid), we used a one-tailed Wilcoxon signed rank test. The alternative hypothesis for this one-tailed test was that the differences between the various dosimetric parameters for photon and proton therapy come from a continuous distribution with a median greater than zero. Differences that were found to be significant at P ≤ 0.05 were then evaluated for significance at P ≤ 0.01. The sequential Bonferroni-type procedure, as described by Benjamini and Hochberg [46], was then used to test for false positives in the independent Wilcoxon sign ranked tests.

## Results

**Figure 2:**
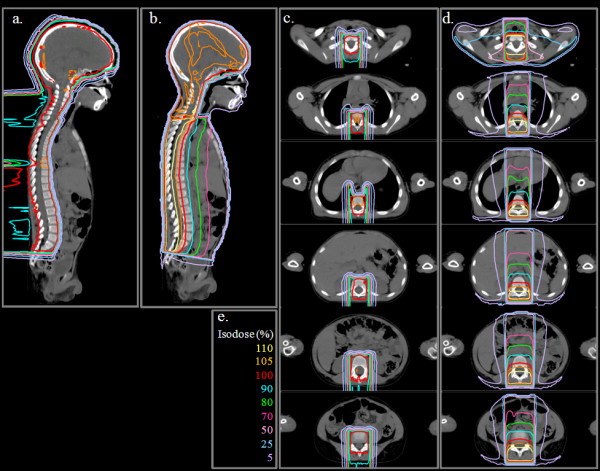
** Photon and proton treatment plans for a representative patient under the age of 15 (this patient was 4 years old, index 2).** (**a**) Proton dose distribution in the sagittal plane. (**b**) Photon dose distribution in sagittal plane. (**c**) Proton dose distribution in axial planes from the cervical spine to the sacral spine in 5-cm increments. (**d**) Photon dose distribution shown in for axial planes from the cervical spine to the sacral spine in 5-cm increments. (**e**) Isodose scale for both photon and proton treatment plans.

**Figure 3:**
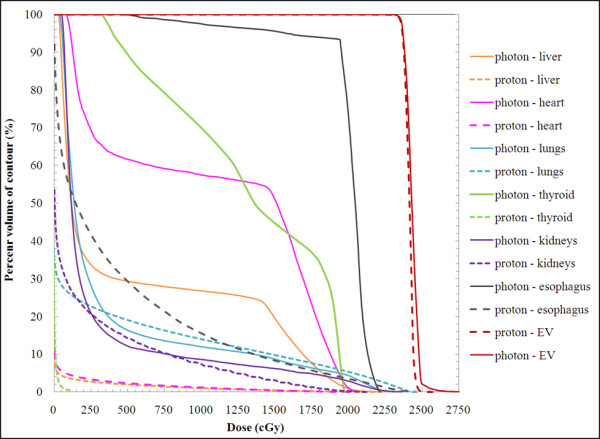
** Photon and proton dose volume histograms (DVHs) for a representative patient (age 4, index 2) under the age of 15.** Proton and photon DVHs are indicated by dashed and solid lines, respectively. The absolute dose values shown on the horizontal axis of 250, 500, 750, 1000, 1250, 1500, 1750, 2000, 2250, and 2750 cGy correspond to percent dose values of 11, 21, 32, 43, 53, 64, 75, 85, 96, 107, and 118%, respectively.

**Figure 4:**
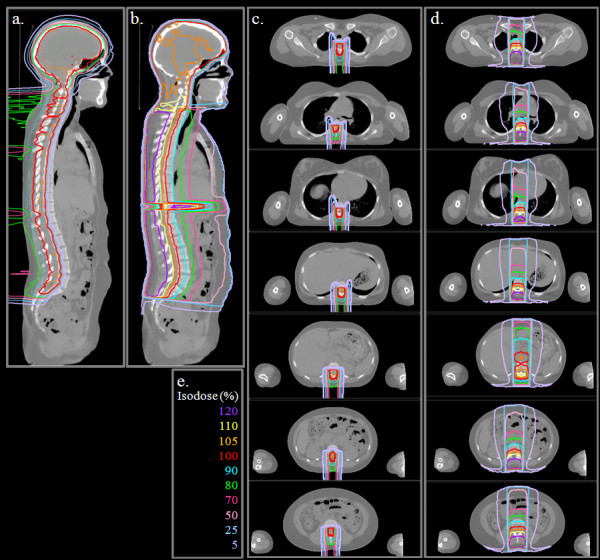
** Photon and proton treatment plans for a representative patient over the age of 15 (this patient was 16 years old, index 13).** (**a**) Proton dose distribution in the sagittal plane. (**b**) Photon dose distribution in the sagittal plane. (**c**) Proton dose distribution in axial planes from the cervical spine to the sacral spine in 5-cm increments. (**d**) Photon dose distribution in axial planes from the cervical spine to the sacral spine in 5-cm increments. (**e**) Isodose scale for both photon and proton treatment plans.

**Figure 5:**
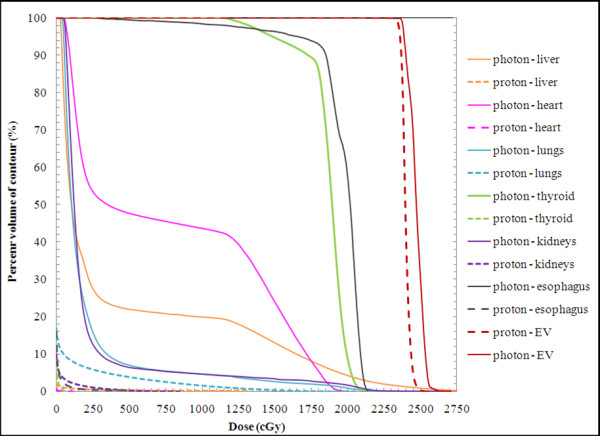
** Photon and proton dose volume histograms for a representative patient (age 16, index 13) over the age of 15.** Proton and photon DVHs are indicated by dashed and solid lines, respectively. The absolute dose values shown on the horizontal axis of 250, 500, 750, 1000, 1250, 1500, 1750, 2000, 2250, and 2750 cGy correspond to percent dose values of 11, 21, 32, 43, 53, 64, 75, 85, 96, 107, and 118%, respectively.

Isodose distributions (Figure 2) and DVHs (Figure 3) for the photon and proton treatment plans for a representative patient under the age of 15 are shown (index 2). In Figure 2, the 100% isodose line indicates the intended treatment region. Qualitatively, several observations can be made: (1) the prescribed dose covers all the vertebral bodies in the proton plan but covers only the spinal canal in the photon plan; (2) the proton dose rapidly decreases beyond the target volume, whereas the photon dose gradually decreases; and (3) the normal organs and tissues in close proximity to the treatment volume receive substantially lower doses from the proton plan than from the photon plan. Isodose distributions (Figure 4) and DVHs (Figure 5) for photon and proton treatment plans for a representative patient over the age of 15 (index 13) are also shown. As in the younger patient, the photon target volume in this patient included the craniospinal canal. However, because this patient was older than 15, the proton target volume was the same as the photon target volume. The qualitative observations for this older patient were similar to those for the younger patient, except that the normal tissue sparing in the proton plan was even greater for this patient because the sharp dose fall-off began at the anterior end of the spinal canal rather than at the anterior end of the vertebral bodies. The dose distributions and DVHs for these two representative patients (ages 4 and 16) highlight the differences in photon and proton dose distributions that result from age-specific treatment volumes.

When comparing the dose distributions in Figures 2 and 4, there is another another age/size effect due to the number of fields required to cover the spinal canal. For the younger patient (Figure 2), the proton and photon treatments could both be delivered using a single spinal field. For the older patient (Figure 4), 3 proton fields and 2 photon fields were required to cover the spinal canal. For both the older and younger patients, the proton dose distributions were homogeneous along the spine, regardless of the number of spinal fields required to treat the spinal canal, whereas the photon dose distribution had hot and cold spots on either side of the spine field junctions when more than 1 spinal field was required (as in Figure 4). While the dosimetric impact of field junctions in CSI was previously known to clinicians, this study highlights the difference in field junction dosimetry between photon and proton CSI.

Quantitative dose-volume results are summarized in Tables 2, 3, and 4 for the photon and proton treatment plans. In the next three subsections, we detail the results from our analysis of the modalities’ coverage of the CTV, sparing of in-field organs, sparing of partially in-field organs, and sparing of out-of-field organs. As mentioned in the methods, that while we chose to use a prescribed dose of 23.4 Gy in this study, prescribed doses as low as 18 Gy and as high at 39 Gy have been used for CSI. Therefore, percent dose is given in parenthesis next to each parameter that is reported in Gy so that our data can be easily translated to any prescription dose. Similarly, percent doses are given in the table captions for each table where absolute doses are reported.

**Table 2:** Dose volume histogram (DVH) analysis for photon and proton craniospinal irradiation (n = 18)

**Structure, DVH dose level (Gy)**	**Photons**		**Protons**		***P*value, Wilcoxian Signed Rank test**	**Significance level (P ≤ 0.05)**
	**Mean**	**SD**	**Mean**	**SD**		
CTV						
23.4	99.36	1.04	99.23	0.88	5.42E-01	NS
25	12.01	9.87	1.72	4.01	8.63E-04	<0.01
Spinal cord						
23.4	99.16	0.85	98.91	1.46	9.83E-01	NS
25	59.02	9.15	4.64	7.52	1.96E-04	<0.01
Optic chiasm						
23.4	100.00	0.00	100.00	0.00	n/a	N/A
25	3.48	4.29	3.26	3.36	5.69E-01	NS
Cochlea						
23.4	99.96	0.16	99.00	3.09	1.56E-02	< 0.05
25	2.08	2.96	4.26	6.95	6.36E-01	NS
Brain stem						
23.4	98.51	3.20	98.96	1.76	8.39E-01	NS
25	0.68	1.39	1.70	7.61	3.12E-02	< 0.05

**Note:** The table provides mean V_23.4_ and V_25_ and standard deviation (SD) values for the CTV and in-field organs for proton and photon therapy. Statistical results from Wilcoxon signed rank test (i.e., two-tailed P-value for Wilcoxon signed rank test) are also listed.

For this study 23.4 and 25 Gy is equivalent to 100% and 106.8% of the prescribed dose, respectively.

**Table 3:** Comparison of parameters to evaluate dose variation with in the target for photon and proton craniospinal irradiation (n = 18)

**CTV dosimetric parameters**	**Photons**				***P*value, Wilcoxian Signed Rank test**	**Significance level**
	**Mean**	**SD**	**Mean**	**SD**		
CI	0.99	0.01	0.99	0.009	5.28E-01	NS
HI	1.05	0.009	1.04	0.012	2.47E-03	<0.01
D_max_	28.13	15.21	26.05	7.868	1.96E-04	<0.01

**Table 4:** Dose volume histogram (DVH) analysis for photon and proton craniospinal irradiation (n = 18)

**Structure, DVH dose level (Gy)**	**Photons**		**Protons**		***P*value, Wilcoxian Signed Rank test**	**Significance level**	**Sequential Bonferroni**
	**Mean**	**SD**	**Mean**	**SD**			
Esophagus							
20	65.87	23.54	3.89	7.68	9.82E-05	<0.01	†
15	96.68	5.12	8.73	12.53	9.80E-05	<0.01	†
10	98.09	3.26	14.76	16.59	9.75E-05	<0.01	†
5	99.61	0.98	24.67	21.28	9.80E-05	<0.01	†
Heart							
20	2.80	4.58	0.03	0.08	2.51E-04	<0.01	†
15	42.49	16.98	0.15	0.23	9.82E-05	<0.01	†
10	56.77	11.12	0.53	0.62	9.82E-05	<0.01	†
5	60.68	11.30	1.31	1.28	9.82E-05	<0.01	†
Kidneys							
20	2.03	1.91	0.60	0.82	3.15E-03	<0.01	†
15	4.11	2.81	2.49	2.34	1.07E-03	<0.01	†
10	5.92	3.68	5.53	4.70	2.50E-01	NS	—
5	8.89	4.95	10.58	7.95	6.24E-01	NS	—
Liver							
20	3.09	3.17	0.08	0.15	9.82E-05	<0.01	†
15	14.69	4.22	0.27	0.30	9.82E-05	<0.01	†
10	22.55	1.99	0.61	1.05	9.82E-05	<0.01	†
5	24.78	3.95	1.10	0.75	9.82E-05	<0.01	†
Lungs							
20	3.07	2.14	2.27	1.92	3.54E-02	< 0.05	*
15	6.03	2.92	4.87	3.24	6.14E-03	< 0.01	†
10	8.35	3.52	7.66	4.32	7.23E-02	NS	—
5	11.69	4.51	11.31	5.52	1.53E-01	NS	—
Thyroid							
20	11.91	21.19	0.00	0.00	6.10E-05	<0.01	†
15	66.16	30.19	0.00	0.00	9.80E-05	<0.01	†
10	80.97	21.53	0.00	0.00	9.48E-05	<0.01	†
5	92.50	10.68	0.51	0.76	9.65E-05	<0.01	†

Note: The table lists mean V_20_, V_15_, V_10_, and V_5_ and standard deviation (SD) values for the esophagus, heart, kidneys, liver, lungs, and thyroid. Statistical results from the Wilcoxon signed rank test (i.e., p-value for 1-tailed Wilcoxon signed rank test) are listed. Results from the sequential Bonferroni procedure are also included; † and * indicate differences were significant at the 0.01 and 0.05 levels, respectively.

For this study 5, 10, 15, and 20 Gy are equivalent to 21.4%, 42.7%, 64.1%, and 85.5% of the prescribed dose, respectively.

### CTV coverage

No significant difference was observed between the photon and proton plans in the mean values of the V_23.4(100%)_ for the CTV (Table 2). For both modalities, the mean V_23.4(100%)_ value was greater than 99%. Similarly, no significant difference in the CI was observed between photon and proton treatment plans, which was greater than 0.99 for both modalities, indicating that the dose distribution conformed well to the CTV (Table 3). In contrast, statistically significant differences were observed in the D_max_, V_25(107%)_, and HI values (Tables 2 and 3). Both the mean D_max_ (P = 1.60E-05) and mean V_25(107%)_ values (P =1.04E-03) were greater for the photon plans, indicating higher maximum doses and higher doses to a larger percentage of the volume. The mean HI was greater for the photon plans than the proton plans (P = 4.87E-04), indicating that the photon dose distributions were more heterogeneous than the proton dose distributions. In summary, the photon and proton treatment plans both provided very good coverage and conformed well to the craniospinal axis, but in general, the photon plans were (approximately 8%) hotter than the proton plans.

### Tissue sparing of in-field organs

The cochleae, brainstem, spinal cord, and optic chiasm were entirely within the 100% isodose region in the photon and proton plans for all patients. We observed no significant difference between the mean V_23.4(100%)_ values from the photon and proton plans value for the brainstem and spinal cord. For the cochleae, there was a significant difference (P = 1.56E-2) in the mean V_23.4(100%)_ values, with the mean photon plan value being approximately 1% greater than for the mean proton plan value (99.96 ± 0.16% for photons versus 99.00% ± 3.09% for protons, P = 5.57E-14, Table 2). No significant difference between the mean V_25(106.8%)_ values from the photon and proton plans value for the optic chiasm and spinal cord. In addition, for brainstem the mean V_25(106.8%)_ value was lower in the photon plans than in the proton plans (P = 3.12E-02) but the mean values for both treatment techniques were less than 2% (0.68 ± 1.39% for photons versus 1.70% ± 7.61% for protons, P = 5.57E-14, Table 2). In contrast, for the spinal cord the mean V_25(106.8%)_ value was much higher in the photon plans than in the proton plans (59.0% ± 9.2% for photons versus 4.6% ± 7.5% for protons, P = 5.57E-14, Table 2). The spinal cord was part of the CTV and these data parallel those that were observed for the CTV, i.e., photon plans resulted in more heterogeneous dose distributions and had larger hot spots than the proton plans.

### Tissue sparing of partially in-field organs and out-of-field organs

In summary, we evaluated 24 individual dosimetric parameters (V_5(21.4%)_, V_10(42.7%)_, V_15(64.1%)_, and V_20(85.5%)_) for six partially in-field and out-of-field organs). The Wilcoxon sign ranked test results indicated that 20 of the 24 parameters (83%) had effects that were significantly different between the proton and photon treatments at the 0.05 level, Table 4. Results of the sequential-type Bonferroni procedure were consistent with those from the Wilcoxon sign ranked tests and did not find any false positives.

Results for individual organs are summarized in Table 4. For the esophagus, heart, liver, and thyroid, there was a significant difference observed between the photon and proton plans for V_5(21.4%)_, V_10(42.7%)_, V_15(64.1%)_, and V_20(85.5%)_ with the values all being higher for photons than for protons. For the kidneys and lungs, there were significant differences observed between the photon and proton plans for V_15(64.1%)_, and V_20(85.5%)_, again with the values higher for photons than for protons. However, a similar difference was not observed at the lower dose levels of 5 and 10 Gy (21.4% and 42.7%).

## Discussion

In this study, we compared proton and photon CSI for 18 patients. It is important to note that this cohort included both male and female medulloblastoma patients whose ages, heights, and weights spanned a clinically relevant and representative spectrum (age 2–16, BMI 16.4–37.9 kg/m^2^) and that we compared the current standard of care at our institution (UTMDACC) for each modality. Furthermore, our patient cohort is the largest, to date, in which CSI with proton and photon therapies have been compared, a feature that constitutes this study’s major strength. Finally, this study addressed differences in the various dosimetric parameters associated with variations in target volume definition, (i.e., that proton volumes were age-dependent, whereas photon target volumes were the same for all patients). In the end, we found that proton CSI improves normal tissue sparing while also providing more homogeneous target coverage than photon CSI for patients across a wide age and BMI spectrum.

For this population of patients, we found that proton CSI provided similar CTV coverage to that of photon CSI but allowed for a statistically significant reduction in doses to non-target organs in close proximity to the craniospinal axis. Moreover, proton treatment plans had greater dosimetric homogeneity along the craniospinal axis than photon treatment plans. Our results thus indicate that proton CSI is superior to photon CSI over the entire age range of children and adolescents affected by medulloblastoma. These results are consistent with those from earlier studies of fewer patients [30-33].

The differences that were observed between the photon and proton treatment plans were primarily due to the differences in the physical properties of photon and proton beams and the physical location of the organs relative to the intended target volume. The esophagus, heart, and thyroid were anterior to the treatment volume and thus were located in a high dose gradient for the photon plans, leading to a higher percentage of the structures receiving 5, 10, 15, and 20 Gy (21.4%, 42.7%, 64.1%, and 85.5%). In contrast, for the proton plans, these organs were beyond the distal edge of the Bragg peak, leading to a substantially lower percentage of the organs receiving 5, 10, 15, and 20 Gy (21.4%, 42.7%, 64.1%, and 85.5%). The kidneys and lungs are bilateral organs situated to the right and left of the spinal fields. They received higher dose from the proton plans compared to the organs that were anterior to the target volume due to the lateral margins used for planning. This effect was more pronounced for the younger patients (Figure 2), whose treatment volumes included the entire vertebral bodies and whose proton plans required greater distal margins. As a consequence of the lateral and distal margins we observed that similar percentages the kidney and lung volumes receiving 5 and 10 Gy (21.4% and 42.7%) for proton and photon CSI. Like the lungs and kidneys, part of the liver is also lateral to the spinal field, but it is not a bilateral organ. Therefore, compared to the lungs and kidneys, a smaller percentage of the liver volume received 5 and 10 Gy (21.4% and 42.7%) in the proton plans than in the photon plans.

Recently, Brodin et al. [38] reported differences between photon and proton CSI plans for 10 patients whose ages also spanned the range of medulloblastoma patients. However, they considered intensity-modulated proton therapy (IMPT), volumetric-modulated arc photon therapy (VMAT), and conventional photon therapy without modulation. Their findings are limited in their clinical meaningfulness, however, because neither IMPT nor VMAT is routinely used for CSI, and conventional photon therapy has very heterogeneous dose distributions compared to the field-in-field photon therapy technique studied here. Another advantage of our work is that we considered current standards of care for photon and proton therapies that are currently in use. Thus, our findings are directly relevant to clinicians who have the option of treating patients with photon or proton CSI. Despite the differences in study design, there is consistency between the major findings of our study and those of Brodin et al., i.e., that proton CSI improves normal tissue sparing while also providing more homogeneous target coverage than photon CSI.

One limitation of this study is that we only focused on therapeutic dose and did not consider stray dose. For photon therapy, the stray dose would comprise only photons (patient scatter and scatter/leakage from treatment head) because all the treatment plans used beams with an energy of 6 MV, which is below the threshold for photoneutron production. In a previous study, we examined the accuracy of the TPS used in this study to predict dose outside of the treatment field, where stray dose is the main component; we found that the TPS was accurate at doses of approximately 5% or more of the prescribed dose [47], which would be 1.17 Gy in the present study, with its prescribed dose of 23.4 Gy. The lowest dosimetric parameter considered here was the V_5_, and the photon dose at this level was accurate, as reported by the TPS. For proton therapy, stray dose is composed almost entirely of secondary neutrons. Dose from stray neutrons was not calculated by the TPS. However, previous Monte Carlo studies [13,48,49] have reported neutron organ doses (for the same proton treatment apparatus used in this work) between 0.83 and 61 mSv/Gy for proton CSI, which in this study corresponds to between 0.0194 Sv and 1.43 Sv for the prescribed dose of 23.4 Gy. As discussed above, the lowest dosimetric parameter considered here was the V_5_, and inclusion of the stray neutron doses would not have changed the V_5_ values. Therefore, neglecting stray neutron dose was not a serious limitation of this study. Nevertheless, stray dose would be an important component of a full comparison of photon and proton therapy for CSI, especially for stochastic late effects such as second cancers, and is therefore part of our ongoing research in radiogenic late effects.

## Conclusions

In conclusion, this study demonstrated that proton CSI improved normal tissue sparing while also providing more homogeneous target coverage than photon CSI. Although the present study focused on medulloblastoma, our findings are generally applicable to other tumors that are treated with CSI. Future work should calculate organ equivalent doses, which include both therapeutic and stray doses.

## Competing interests

The authors declare that they have no competing interests.

## Authors’ contributions

RH conceived of the study, drafted (and edited) the manuscript, participated in creating the FIF photon plans, and performed the statistical analysis. AG created the proton plans and helped draft the manuscript. WK created the photon FIF treatment plans and specifically was involved in adapting the CSI FIF technique for the TPS used in this study. AM provided guidance on treatment plan design and reviewed all treatment plans, and reviewed/edited the manuscript. WN assisted with reviewing/editing the manuscript and provided funding for the study. CE provided guidance for the statistical analysis and reviewed all statistical results in detail. AD participated in the statistical analysis and wrote code to perform the Bonferroni procedure. KH wrote codes to extract DVH data from TPS and format data for statistical analysis; he also assisted with data analysis and in reviewing manuscript. All authors read and approved the final manuscript.
